# Structure and function of the kidney in septic shock - a prospective controlled study

**DOI:** 10.1186/2197-425X-3-S1-A838

**Published:** 2015-10-01

**Authors:** MJ Maiden, S Otto, J Brearly, MJ Chapman, CH Nash, J Edwards, TR Kuchel, R Bellomo

**Affiliations:** Royal Adelaide Hospital, Intensive Care Unit, Adelaide, Australia; Acute Care Medicine, University of Adelaide, Adelaide, Australia; SA Pathology, Adelaide, Australia; SAHMRI, PIRL, Adelaide, Australia; Austin Hospital, Intensive Care Unit, Heidelberg, Australia

## Introduction

Sepsis is the most common cause of acute renal failure. Impaired renal blood flow, acute tubular necrosis, glomerular micro-thrombi and apoptosis have all been proposed to cause this renal dysfunction [1,2]. However, these concepts have been largely based on uncontrolled, observational and post-mortem studies.

## Objectives

Determine changes in renal structure and function over time in an ovine model of septic shock managed with intensive care support.

## Methods

Fifteen sheep had renal artery flow probes and renal vein cannula inserted. Ten sheep were given intravenous *E.coli* to induce sepsis and five acted as non-septic controls. Animals were managed for 48 hours with protocol guided ventilation, sedation, parenteral fluids and noradrenaline (NorA) infusion to maintain mean arterial pressure 75mmHg. Renal biopsies were taken from all animals at baseline, 24 and 48 hours or if oliguric for more than two hours. Biopsies were analysed under light and electron microscopy and appearance systematically quantified by expert renal pathologists blinded to group allocation and time of biopsy.

## Results

Sheep given *E.coli* developed hyperdynamic septic shock requiring NorA infusion. Renal blood flow was not different between septic and non-septic groups over the duration of the study (mean ± SD, mL/min; non-septic 428 ± 102 vs. septic 386 ± 118; p = 0.12). Septic sheep became oliguric and after 48 hours had increased serum creatinine (mmol/L; non-septic 69 ± 14 vs. septic 200 ± 86; p < 0.01) and reduced creatinine clearance (mL/min; non-septic 170 ± 44 vs. septic 40 ± 40; p < 0.01). There was no difference in mean renal O_2_ consumption (mL/min; non-septic 4.9 ± 2.1 vs. septic 5.6 ± 2.3; p = 0.39). Histopathological and ultrastructural appearance of each component of the nephron did not change over time and was not different between the septic and non-septic group of animals.

## Conclusions

In this ovine model of Gram-negative septic shock, acute renal dysfunction was not associated with changes in renal blood flow, O_2_ consumption, cellular appearance or ultrastructural change. Other mechanisms are likely to contribute to the acute changes of renal function in sepsis.

## Grant Acknowledgment

Intensive Care Foundation University of Adelaide, Maurice Sando Research Grant
